# Relationship between the Expression of the Thymidylate Synthase and the Prognosis of Gastric Cancer Patients Treated with Combinational Chemotherapy Regimen Including Fluorouracil, Docetaxel and Cisplatin 

**DOI:** 10.18502/ijhoscr.v14i3.3727

**Published:** 2020-07-01

**Authors:** Mozaffar Aznab, Seyed Majid Ahmadi, Sedigheh Khazaei, Seyed Mojtaba Ahmadi, Mansour Khazaei, Hamid Hamdi

**Affiliations:** 1 Department of Internal Medicine, Taleghani Hospital, Kermanshah University of Medical Sciences, Kermanshah, Iran; 2 School of Medicine, Yasuj University of Medical Sciences, Yasuj, Iran; 3 Molecular Pathology Research Center, Imam Reza Hospital, Kermanshah University of Medical Sciences, Kermanshah, Iran; 4 Department of Clinical Psychology, School of Medicine, Kermanshah University of Medical Sciences, Kermanshah, Iran; 5 Taleghani Hospital, Kermanshah University of Medical Sciences, Kermanshah, Iran; 6 Army Hospital, Sanandaj, Kurdistan

**Keywords:** Thymidylate synthase, Gastric cancer, 5- fluorouracil, Survival, Chemotherapy

## Abstract

**Background:** Thymidylate synthase is one of the target enzymes of 5-fluorouracil. However, the clinical and prognostic significance of TS expression in gastric cancer has remained controversial. In this study, the expression of thymidylate synthase was evaluated in gastric cancer patients treated with combinational chemotherapy; moreover, the association between TS expression and clinicopathologic characteristics and overall survival of the patients were also assessed.

**Materials and Methods:** In this descriptive study, 89 pathological samples were gathered from patients at Kermanshah hospitals during 2008-2017. The survival status of patients was recorded and their overall survival was evaluated individually.

**Results:** The average survival period for low and high thymidylate synthase groups was 54 and 50 months, respectively, meaning higher survival time in the lower thymidylate group. But this difference was not statistically significant (log Rank=0.88). In addition, sex, stage, recurrence, and survival had no significant difference between the low and high expression of thymidylate synthase groups (p=0.89).

**Conclusion:** The results clearly indicated that the level of thymidylate synthase is not a significant modulator of 5- fluorouracil in gastric cancer patients. Nevertheless, evaluation of the level of the enzymes and markers as well as their effects are highly recommended for accurate selection of chemotherapeutical strategies.

## Introduction

 As one of the high-prevalence cancers, gastric adenocarcinoma has been recognized as one of the major death causes^1^. In the United States, 28,000 out of 100,000 people were estimated to be diagnosed with stomach cancer in 2019^2^. It has been well known that gastric cancer is associated with several factors including geographical location, diet, host genetic factors, and other pathogens. Owning to the genetic variation and various lifestyles, the rate of gastric cancer varies across different regions of the world^3^. In 2005, the highest frequency of gastric cancer was reported in Japan, China, and Russia; while the least frequency was recorded in the developed countries^4^^, ^^5^. Comparing the incidence of stomach cancer in different parts of the world, this disease has a high prevalence in Southeast Asia and possesses a low incidence in North America^6^^,^^7^. In general, the prevalence of this type of cancer in developing countries is higher than the developed countries^7^. The incidence of gastric cancer remains unchanged among first-generation immigrants with the same resident risk suggesting the effect of environmental communication and carcinogenic food on the development of gastric cancer^2^^, ^^8^. After two generations, this outbreak is similar^9^. Among the risk factors, Helicobacter pylori infection ^10^^ ,^^11^^ ,^^12^, high intake of salty and smoked foods^13^, salt-preserved foods^13^, smoking, low socioeconomic status, low fruits and vegetable consumption, gastro-esophageal reflux disease (GERD) and male sex can be mentioned^13^. Thus, gastric cancer could be classified as sporadic and/or inherited disorder. Over the last 20 years in developed countries, the incidence of gastric cancer has decreased in the distal stomach, while the proximal stomach cancer exhibited an increase^14^. This may be due to a reduction in the H. pylori infection. The five-year survival of patients with distal stomach cancer at stage I/II is reported to be 50%, for patients with proximal gastric cancer this parameter was, however, 10% - 15%. Due to the lack of a distinctive biomarker in initial stages, gastric cancer is unfortunately diagnosed at advanced stages with poor prognostic markers. Despite determining factors for the relapse and survival of gastric cancer (such as the TNM system), it is not yet possible to accurately predict the relapse and survival of patients with gastric cancer. There are patients whose clinicopathologic manifestations have not been accurately assessed for high recurrence probability, who eventually experienced recurrence and died. It is necessary to provide better treatment to the majority of the patients suffering from the progression of the disease after several months following first- and second- line treatments. Several therapeutic strategies have been considered to improve the survival of patients with gastric cancer; neoadjuvant chemotherapy (magic trial)^15^, chemoradiotherapy after surgery (trial itergroup0116)^16^, and surgery with adjuvant chemotherapy^17^^,^^18^ to name a few. Different treatment regimens have been used to treat gastric cancer. Some of these regimens employ the newer generation of target therapy, which has not worked well. 5- FU is the most important drug used in this approach. There is no proper molecular biomarker to predict the effect of 5FU. Synthetic thymidylate is the critical target for 5FU. It has been suggested that 5-FU can inhibit cellular proliferation through irreversible inhibition of thymidylate synthase. Thymidylate synthase (TS) is a unique enzyme involved in the conversion of dUMP to dTMP as well as the synthesis of DNA ^19^. In this regard, TS inhibition leads to an imbalance in the levels of deoxynucleotides and increases the content of dUMP that is associated with DNA damages. Therefore, TS seems to be the main target for the chemotherapeutical agent which could develop drug-resistance^20^. Several studies have associated the TS expression levels with chemotherapy response in patients receiving a 5-FU baseline regimen. Furthermore, low TS expression has been reported to increase survival. In some clinical studies, increased TS expression developed 5FU-resistance. The expression of thymidylate synthase and its correlation with the development of cancer has been addressed in several studies ^21^. TS-inhibitors have been proposed as important drug targets. Taken together, the purpose of the present study is to evaluate the expression of thymidylate synthase using the IHC method and explore its correlation with the patients’ survival and their response to 5FU-based chemotherapy regimens.

## MATERIALS AND METHODS

 In this descriptive-analytic study, 89 gastric tumor specimens were sampled from patients treated with a combinational chemotherapy regimen containing 5-fluorouracil (in the time range of 2008 to 2017). The prepared slides from paraffin blocks were stained with the usual Hematoxilin–Eosin methods and then observed by two pathologists to confirm the initial diagnosis. Immunohistological staining was performed on tissue sections using monoclonal antibody against TS. Pathological blocks of the gastric cancer patients were selected for staining after complete deparaffinization. They were then diluted with graded ethanol. Peroxidase activity was inactivated by 3% hydrogen peroxide in methanol for 30 min; the sections were then washed with water, heated in citrate buffer inside a pressurized cooker. After preparing the slides, they were left at room temperature to be cooled in soaked solutions for 30 minutes. Sections were then incubated at room temperature using monoclonal antibodies which interacted with thymidylate synthase. Histophin stain was used as a second layer reagent according to the Global Polymer Immunoboxidase method. Negative control studies were performed without the use of primary antibodies. These stains were observed by two pathologists. The thymidylate synthase expression classification relied on two bases: 1- Staining intensity (low-intensity: scores of zero and one; high-intensity: scores of 2 + and 3 +). 2- Staining area (focal is positive in less than 25% of cells or diffuse is positive in more than 25% of tumor cells). We used the first classification. The overall survival, disease-free survival and their interplay with the expression of thymidylate synthase were studied.

**Statistical analyses**

The data were analyzed by descriptive statistical methods such as t-test, Chi-square, and Kaplan–Meier methods using SPSS (V.18).

## Results

**Figure 1:**
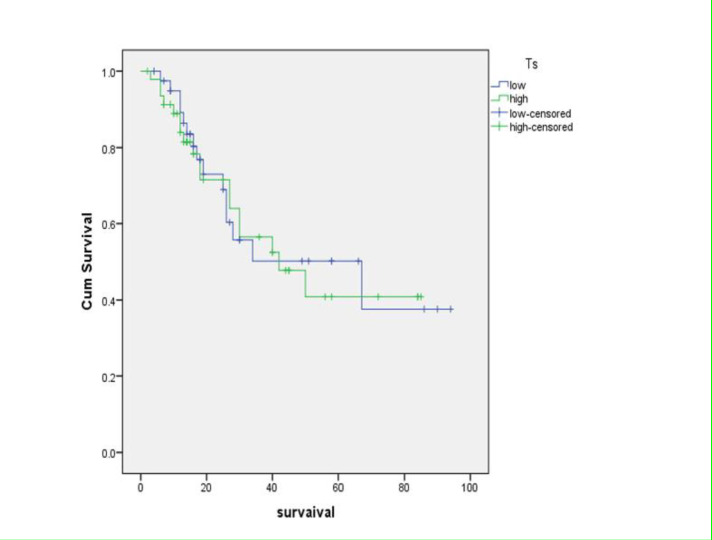
Overall survival of gastric cancer patients according to TS expression

 Of 89 patients that followed their treatment with chemotherapy including 5 fluorouracil from retrieved files, 19 (21.35%) were females and 70 (78.65%) were males. Their ages ranged in 27-80 years with a mean of 57.8± 10.98 years. In addition, 4 (5%) were in stage I, 17 (21.25%) were in stage II, 35 (43.75%) were in stage III and 24 (30%) were in stage IV. Among 89 patients, gastric cancer recurrence was observed in 10 cases. The expression of TS by IHC indicated 25 (28.09%) negative, 16 (17.98%) 1+, 33 (37.08%) 2+, and 15 (16.85%) 3+ results (Table 1). Regarding the previously-mentioned classification, 41 (46.07%) had low and 48 (53.93%) had high TS expression. In total, 34 (38.64%) died and 54 (61.36%) were alive during the study. The survival of patients following diagnosis and treatment was about 3-100 months with a median of 26.79 months. The descriptive characteristics of the patients are summarized in Table 1 based on low and high TS expression. The results showed no significant difference in terms of sex, stage, recurrence and survival between low and high expression of thymidylate synthase groups (p>0.05). T-test results in terms of ages showed no significant difference between the high and low TS expression groups (p>0.05). In spite of the higher survival time for low TS expression (Mean=53.84 and Median=67 months) compared to high TS expression (Mean =49.40 and Median=42 months), no significant difference was detected between the two groups (log rank=0.87) (Figure1).

**Table1:** Demographic and histopathologic characteristics of patients (total n= 89) according to low and high expression of thymydylate synthase

**Variable**		** Low Thymidylate Synthase**	**High Thymidylate Synthase**	**Total**	**sig**
		M±SD	M±SD		
Age		60.02±11.51	55.90±10.24		0.07
		Frequency (%)	Frequency (%)		
sex	male	32(78%)	38(79/2%)	70(78/7%)	0.89
	female	9(22%)	10(20/8%)	19(21/3%)	
stage	1	1(2.6%)	3(7/1%)	4(5%)	0.48
	2	6(15.8%)	11(26.2%)	17(21/3)	
	3	18(47.4%)	17(40.5%)	35(43/8)	
	4	13(34.2%)	11(26.2%)	24(30%)	
regression	yes	3(7.5%)	7(14.6%)	10(11.4%)	0.24
	no	37(92.5%)	41(85.4%)	78(88.6%)	
survival	death	15(37.5%)	19(39.6%)	34(38.6%)	0.84
	live	25(62.5%)	29(60.4%)	54(61.4%)	

## Discussion

 Despite the significant advancements in gastric cancer treatment, this disease has still remained as one of the major health crises. The main challenge is early recurrence which could be attributed to lack of specific and accurate strategies in therapy types and lack of a complete algorithm of prognostic factors to predict its recurrence. New prognostic factors have been recently proposed. The Medical Research Council Adjuvant Gastric Infusional Chemotherapy (MAGIC) trial about gastric cancer showed several prognostic factors including microsatellite stable (MSS) and low or high microsatellite instability ( MSI-L and MSI-H)^22^. A study on an Asian group revealed a number of effective factors on prognosis, microsatellite instability (MSI), microsatellite stable and epithelial-to-mesenchymal transition (MSS/EMT) phenotype, microsatellite stable and presence of TP53 (MSS/TP53+) or no TP53 signature (MSS/TP53-)^23^. These factors may affect the recurrence of disease and the patients’ survival. In some studies, Epstein-Barr virus infection has been implicated in gastric cancer. Some mutations have been also observed in gastric cancer patients as well as Epstein-Barr virus infection including PIK3CA, ARID1A and BCOR mutations^24^. This classification has provided a significant understanding of the molecular and heterogeneous profiles of gastric cancer across different demographics. In addition to genetic changes such as Her2neu amplification, fibroblast receptor amplification/mutation, gross factor fibroblast receptor amplification/mutation, PDL 1/PD1 pathway amplification were listed^20^^,^^26^. These factors have been used as prognostic as well as treatment factors. Some of the drugs applied for these genes or mutations (e.g. Trustozumab) are also used for patients with Her2/neu positive as the first-line treatment. Monoclonal antibody against vascular endothelial gross factor II, called ramosiromab^27^, is used as the second and third line, while pembrolizumab is employed for PDL-expressing tumors as the third line of treatment. Other factors are involved in response to treatment and recurrence of the disease among which is TS. In line with most of the studies, our study showed increased expression (TS) in 60% of the patients. Our analysis also showed 54(Mean=53.84 and Median=67 months) and 50(Mean =49.40 and Median=42 months) months of patients’ survival for the low- and high-expression groups, respectively; whose difference was not statistically significant. In terms of survival rate, our results were similar to the findings of J-H Choi et al.^28^ reporting no significant difference between the patients with high and low expression (TS). However, our results differ with Yeh CN et al. ^29^ studies who found a significant difference between the patients in these two groups. In the study by Yeh CN et al., it was reported that patients with low (TS) expression had longer survival in comparison with those exhibiting high (TS) expression; moreover, most of the high-expression group members were female. In the study by Yeh CN et al., no significant difference was observed in the clinicopathological symptoms of patients with high and low TS expression. But analyses have shown that the patients with low expression responded better to 5FU-based chemotherapy regimen. Most of the patients with low (TS) expression in the tumor were women. Patients with high expression had less recurrence-free survival and overall survival; therefore they concluded that TS expression has an important role in the response to 5FU. In the study by J-H Choi et al., the expression of TS was investigated in patients with advanced gastric cancer who were treated with chemotherapy in terms of its association with clinicopathological and prognosis. In their study, 63% of the patients had a primary tumor with high expression and 37% possessed low expression. Most of the patients with high expression were men. This was similar to our study which reported high TS expression among men. In terms of 4-year survival, there was no significant difference between high and low expression groups. Overall, it seems that TS expression plays an important role in the determination of patients’ survival. It can also determine the response to 5FU chemotherapy. In this regard, the classification of the patients into two groups of low and high expression can help in the determination of treatment type. However, there are still considerable controversies in this regard; and many researchers have not considered an indicative role for TS expression. Most of the studies mentioned the importance of thymidylate synthase screening in gastric cancer patients. Thus, more extensive studies are required among communities of various races. Upon proving the effect of thymidylate synthase as a prognostic factor, it may be recommended to screen it prior to the initiation of the cancer treatment. For patients with high thymidylate synthase levels, resistance to 5FU should be considered and the possibility of concomitant use of other drugs, especially target therapy, should be taken into account. There are other thymidylate synthase inhibitors such as capecitabine. The development of newer generations of thymidylate synthetase inhibitors is also on the road. The limitations of the present study should be also mentioned which can explain the lack of difference in the survival of the two groups. The number of participated patients is probably too small to detect significant differences. The other reason could be also assigned to the pathologically-evaluated samples (in some patients the samples were from endoscopy rather than surgery). It could be also attributed to the cut off considered in pathology staining (we considered above 20% as positive; while many studies have considered 10-50% as positive). The small number of patients or even low experience of the pathologists could be also involved. Moreover, our study employed combinational chemotherapy including 5-Flouracil, docetaxel and cisplatin; 5-FU was also in the form of 24-hour infusion which could be the probable reason for observing no difference between the two groups. 

## CONCLUSION

 The present study revealed that although survival is higher in the group with lower TS expression this difference is not significant. Therefore, TS alone cannot determine disease prognosis, especially among the patients receiving the combinational chemotherapies. However, more research with larger cohorts from multiple centers are required to address the multifactorial etiology of gastric cancer. It is also recommended to investigate other factors to reach a strategy for selecting the proper drug regimen along with targeted therapy.
